# The Transcriptomic and Proteomic Landscapes of Bone Marrow and Secondary Lymphoid Tissues

**DOI:** 10.1371/journal.pone.0115911

**Published:** 2014-12-26

**Authors:** Sandra Andersson, Kenneth Nilsson, Linn Fagerberg, Björn M. Hallström, Christer Sundström, Angelika Danielsson, Karolina Edlund, Mathias Uhlen, Anna Asplund

**Affiliations:** 1 Department of Immunology, Genetics and Pathology, Uppsala University, Uppsala, Sweden; 2 Science for Life Laboratory, Uppsala University, Uppsala, Sweden; 3 Leibniz Research Centre for Working Environment and Human Factors (IfADo) at Dortmund TU, Dortmund, Germany; 4 Science for Life Laboratory, Royal Institute of Technology, Stockholm, Sweden; Rutgers - New Jersey Medical School, United States of America

## Abstract

**Background:**

The sequencing of the human genome has opened doors for global gene expression profiling, and the immense amount of data will lay an important ground for future studies of normal and diseased tissues. The Human Protein Atlas project aims to systematically map the human gene and protein expression landscape in a multitude of normal healthy tissues as well as cancers, enabling the characterization of both housekeeping genes and genes that display a tissue-specific expression pattern. This article focuses on identifying and describing genes with an elevated expression in four lymphohematopoietic tissue types (bone marrow, lymph node, spleen and appendix), based on the Human Protein Atlas-strategy that combines high throughput transcriptomics with affinity-based proteomics.

**Results:**

An enriched or enhanced expression in one or more of the lymphohematopoietic tissues, compared to other tissue-types, was seen for 693 out of 20,050 genes, and the highest levels of expression were found in bone marrow for neutrophilic and erythrocytic genes. A majority of these genes were found to constitute well-characterized genes with known functions in lymphatic or hematopoietic cells, while others are not previously studied, as exemplified by *C19ORF59*.

**Conclusions:**

In this paper we present a strategy of combining next generation RNA-sequencing with *in situ* affinity-based proteomics in order to identify and describe new gene targets for further research on lymphatic or hematopoietic cells and tissues. The results constitute lists of genes with enriched or enhanced expression in the four lymphohematopoietic tissues, exemplified also on protein level with immunohistochemical images.

## Introduction

Since 2003, the Human Protein Atlas (HPA) project has systematically explored the human proteome in a range of normal tissues, cancers and cell lines. The effort is based on a unique set-up of high throughput generation of affinity-purified polyclonal antibodies for *in situ* protein detection using immunohistochemistry (IHC) and immunofluorescence (IF) on carefully designed tissue- and cell microarrays [1]–[3]. The output of the project is a publically available Protein Atlas [4], in which all IHC and IF images along with antibody validation and annotation data are published. In version 12 of the Protein Atlas, data for 21,984 antibodies are included, targeting the protein products of 16,621 unique genes, i.e. 82% of human protein coding genes.

Mapping the human proteome is challenging, as evidence on protein level is missing for more than 30% of the human protein coding genes [5], and consequently a large portion of the human proteome remains unexplored. Several gene expression atlases for global gene expression data on a RNA-level have been launched, such as the Expression Atlas [6], the transcriptional profiling in human and mouse tissues using custom designed Affymetrix chips [7], the centralized gene expression portal BioGPS [8], the repository ArrayExpress [9] and the RNAseq Atlas [10] with transcriptomics data based on deep sequencing of eleven normal human tissue types. These efforts constitute important resources for any project aiming at in-depth analyses of specific genes or at global systems biology studies for an understanding of human biology and disease. Since 2013, the Protein Atlas also includes transcriptomic data from 27 histologically normal tissues, and the extensive data collection on both transcript and protein level has enabled a unique comparative study covering most tissues and cell types in the human body for characterization of housekeeping as well as tissue-specific gene expression patterns [11]. IHC offers a visual representation of protein localization with a cellular spatial resolution in complex tissues, and serves as a valuable complement to global gene expression analyses of transcript levels performed on tissue lysates. The global transcriptomic analysis using deep RNA-sequencing in combination with IHC offers the possibility to identify previously unexplored expression patterns, and to add an additional layer of information regarding cell type and subcellular localization of the expressed protein.

In this article we explore the gene expression profiles in bone marrow, spleen, lymph node and appendix using transcriptomics analysis using next generation deep sequencing (RNAseq). These four tissues, differing in both histology and function, were selected because they have in common the feature of harboring a major cell population of hematopoietic origin, i.e. lymphoid cells. Bone marrow is considered a primary lymphoid organ, and displays the widest range of hematopoietic cells, as it also constitutes the site for hematopoiesis. Secondary lymphoid organs lymph node, appendix and spleen harbor a pronounced population of lymphoid cells, as these are primary sites for e.g. antigen-driven affinity maturation, somatic hypermutation and immunoglobulin class switch of bone marrow derived naïve B-lymphocytes in germinal centers, and also proliferation. In appendix, the mucosa-associated lymphoid tissue (MALT) contains areas of lymphoid tissue encountering antigens passing through the glandular epithelium. As such, appendix is expected to harbor a wide range of cell types, since although the lymphoid tissue consists mostly of B- and T- lymphocytes, it also includes a glandular epithelium and smooth muscle.

The aim of this study was to define the expression profiles of four lymphohematopoietic tissues with a common component of lymphoid cells, and to potentially identify previously uncharacterized proteins within these tissues. The results do not by themselves provide deeper understanding of gene functions, but offer an important foundation for insight into possible mechanisms of identified proteins.

## Results and Discussion

### Overall characterization of the lymphohematopoietic transcriptome

RNAseq analysis of 20,050 protein-coding genes was performed using Illumina HiSeq2000 and 2500 (Illumina) on 95 samples representing 27 different tissue types [11] listed in S1 Table. Overall FPKM (fragments per kilobase of exon model per million mapped reads) values ranged from 0.1 to 59,629, and 17% (3,499) of the 20,050 genes were classified as displaying a tissue- or group enriched expression pattern (see table 1 for definitions of gene expression categories) in one or more of the 27 tissues [11]. The remaining 83% (16,551) displayed a more general expression pattern, categorized as “expressed in all”, or “mixed”, or were classified as “not detected” (see table 1 for definitions of gene expression categories) [11].

**Table 1:** Classification of gene expression patterns as determined over 27 tissues.

Classification of expression pattern	Criteria for classification
Not detected	FPKM level below 1 in bone marrow, lymph node, spleen and appendix
Highly tissue enriched	50-fold higher FPKM level in one tissue compared to the second highest of all other tissues
Moderately tissue enriched	5-fold higher FPKM level in one tissue compared to the second highest of all other tissues
Group enriched	5-fold higher average FPKM level within a group of 2–7 tissues including at least one tissue of lymphohematopoietic origin compared to the second highest of all other tissues.
Enhanced	5-fold higher FPKM level in one tissue compared to the mean FPKM in all other tissues together
Expressed in all	FPKM level above 1 in all tissues
Mixed	Genes expressed in 1–26 tissues and not according to any of the criteria described above

This article focuses on the expression profile in four tissues (bone marrow, spleen, lymph node and appendix) harboring a major component of cells of hematopoietic and lymphatic origin. In these four tissues, FPKM values ranged from 0.1 to 25,947, and the maximum FPKM value for each tissue type is indicated in table 2. Altogether 693 genes displayed an enriched or enhanced expression in one or more of the lymphohematopoietic tissues. 54% (10,869) of all genes were found to be expressed in all four lymphohematopoietic tissues. Out of these 10,869 genes, 85% (9,222) were also found to be expressed in all other tissue types, suggesting that they perform “housekeeping” functions.

**Table 2:** Summary of transcriptomic profiling of lymphohematopoietic tissues.

Tissue (# biol. replicates)	% genes expressed	Top FPKM value	Correlation between biological replicates
Bone marrow (4)	57	25947	0.95–0.97
Lymph node (5)	65	5439	0.95–0.97
Spleen (4)	66	5524	0.98
Appendix (3)	69	5365	0.96–0.98

### Comparative analysis between lymphohematopoietic tissues

**Figure 1:**
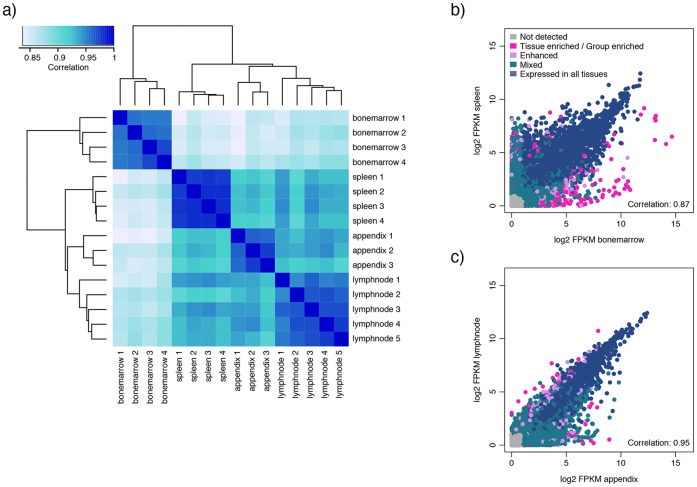
Dendrogram and correlation plots for the lymphohematopoietic tissues. a) Dendrogram covering all samples of the four lymphohematopoietic tissue types visualizing clustering according to gene expression profiles. Correlation plots are shown for b) spleen and bone marrow displaying the lowest correlation between the four lymphohematopoietic tissues, and for c) lymph node and appendix displaying the highest correlation, based on the average FPKM-value of all samples within the same tissue type (n = 3–5).

Three to five samples for each tissue type were analyzed, and the correlation between biological replicates was high (0.95–0.98; Table 2). Spleen constitutes the tissue with the highest correlation between biological replicates, with Spearman correlation coefficients of 0.98 for all combinations. This is also evident from Fig. 1a, in which the dendrogram and the color codes reveal that the biological replicates for each tissue type cluster together, being more similar to each other than to those of the other tissues types, and that spleen samples display the most similar expression profiles.

From the dendrogram (Fig. 1a) it is evident that bone marrow samples share an expression profile that is distinct from the other three tissues types. At the next level of separation, appendix and lymph node diverge from spleen, and bone marrow and spleen are the two tissues that are furthest away from each other and differ the most in respect to their expression profiles, with a correlation coefficient of 0.87 (Fig. 1b).

The two most similar tissues are lymph node and appendix, with a correlation coefficient of 0.95 (Fig. 1c). In the correlation plot for appendix and lymph node it is evident that most of the transcriptome is shared between the two tissues, and that very few genes display a lymph node characteristic expression compared to appendix. Appendix, on the other hand, displays expression of a number of genes (78) that are not expressed in lymph node. More than half of these (53%) are also expressed in colon and/or small intestine (including duodenum), suggesting a function related to the GI tract. Examples of these genes are *CEACAM5* and *GAL*. *CEACAM5*, better known as carcinoembryonic antigen (*CEA*), is a glycoprotein and is used as a diagnostic biomarker for several cancer types, e.g. breast and colon cancer [12]. IHC staining of the protein CEACAM5 shows membranous and cytoplasmic staining mainly located to the GI-tract, and even stronger staining in colorectal cancer (S1 Fig.). *GAL* codes for a small neuropeptide that functions as a cellular messenger within the central and peripheral nervous systems [13]. Protein expression of *GAL* was confirmed by IHC staining of ganglion in e.g. colon (S1 Fig.). The other 37 genes uniquely expressed in appendix compared to lymph node did consequently not show an elevated expression in other GI tissues. Gene ontology (GO)-analyses of these 37 genes reveal an overrepresentation of genes involved in immune response, inflammatory response and chemotaxis. *CXCR1* and *CXCR2* encode two receptors for neutrophil chemotactic factor IL-8, and both show an enhanced expression in appendix. This is indicative of an active inflammation, as the proteins CXCR1 and CXCR2 act as chemotactic agents that activate neutrophils to migrate to sites of inflammation [14]. All samples of appendix in this study represent cases of appendicitis, and an active inflammation may very well signify the difference in gene expression profiles compared to other GI-tissues and lymph node.

### Transcriptomics defining different hematological tissues

**Figure 2:**
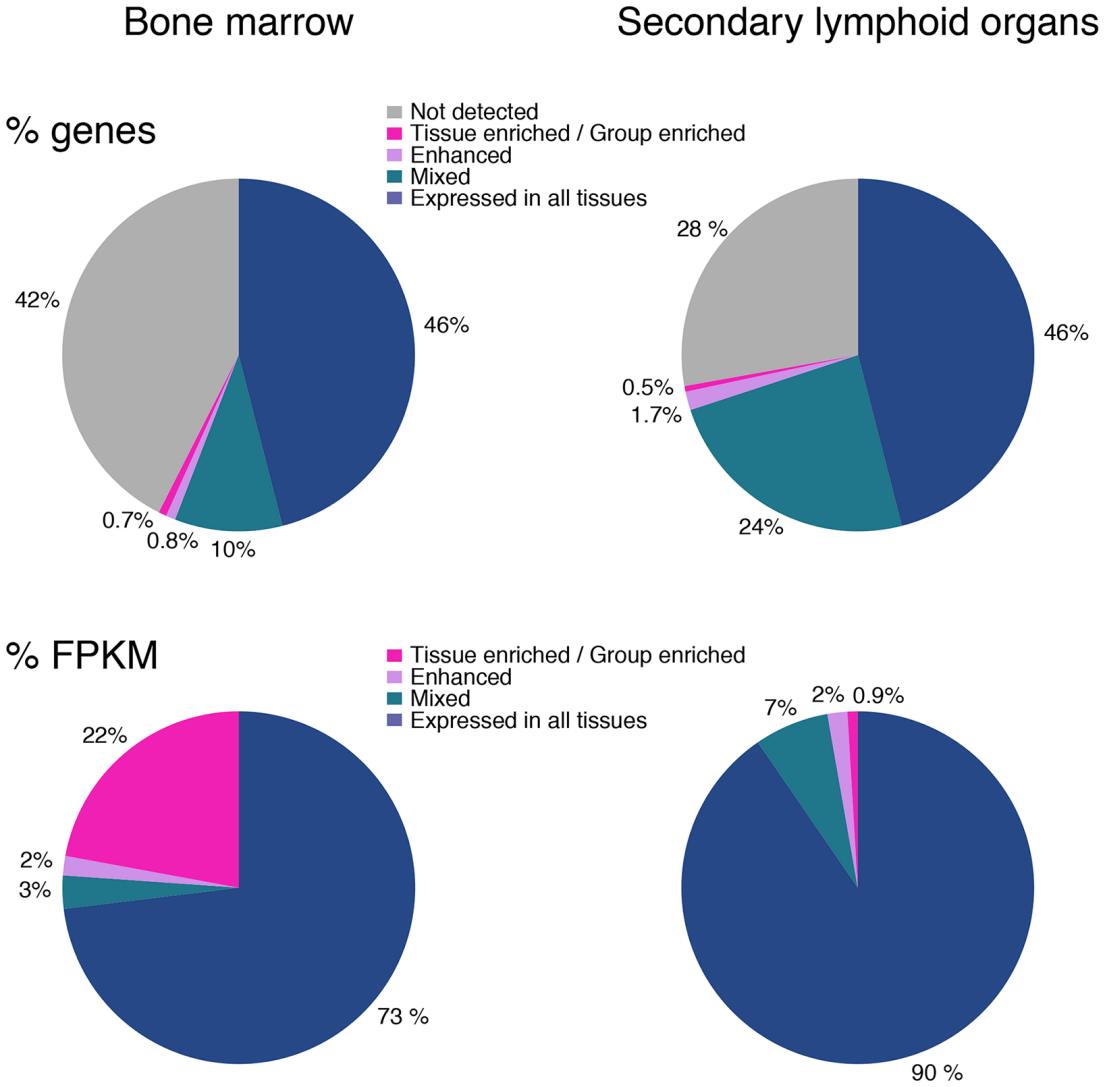
Pie charts displaying the expression profiles for the lymphohematopoietic tissues. The upper panel shows the percentage of genes expressed within the different categories of expression, for bone marrow and for the secondary lymphoid organs respectively. The lower panel pie charts show the percentage of transcripts (FPKM) for each category.

The pie charts in Fig. 2 display the representation of gene expression categories (listed in table 1) in the primary lymphoid organ bone marrow (left) and in the secondary lymphoid organs lymph node, spleen and appendix (right). The color codes visualize the fact that the fraction of genes *not detected* is greater in bone marrow as compared to the secondary lymphoid tissues; perhaps not surprising, considering that the three secondary lymphoid tissues together harbor a greater range of cell types and consequently a larger variety of expressed genes. In support of this, appendix displays fewer genes *not detected* (S2 Fig.) than any of the other lymphohematopoietic tissues, possibly due to the presence of diverse cell types such as glandular epithelium, smooth muscle and lymphoid cells. Ficoll separated bone marrow, on the other hand, represents a relatively homogenous population of hematopoietic cells, from which mature erythrocytes, granulocytes, bone marrow stromal cells, adipocytes, and cells of vessels have been removed.

The number of tissue/group enriched genes (pink) is low both for bone marrow, 137 (0.7%), and secondary lymphoid tissues, 104 (0.5%), suggesting that a large portion of the transcriptome is shared among several tissues. The corresponding numbers of enhanced genes (5x higher FPKM than mean FPKM in all other tissues) were 164 (0.8%) in bone marrow and 340 (1,7%) in the secondary lymphoid organs.

When taking into account the number of detected transcripts from each gene (Fig. 2), it becomes very evident that the small fraction of tissue/group enriched genes expressed in bone marrow are expressed at very high levels, as they make up for almost 25% of the total number of transcripts in bone marrow. The nine tissue enriched genes with the highest expression in bone marrow code for proteins with known functions in neutrophils (*DEFA1*, *DEFA1B*, *DEFA3*, *DEFA4*, *CTSG* and *MPO*) and erythrocytes (*HBB*, *HBA1* and *HBA2*; all three codes for hemoglobin proteins), both of which reach maturity in bone marrow and are released into the blood stream as effector cells, equipped with necessary proteins for their specialized functions. Consequently, a high level of transcription of these genes takes place in bone marrow, explaining the high FPKM-values. This is also evident in table 2, in which the range of FPKM values is much greater in bone marrow than in the other three tissues.

**Figure 3:**
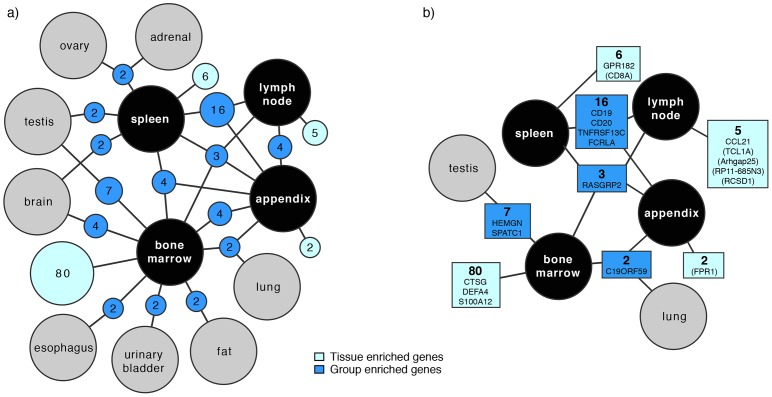
Network plot visualizing the relationship of enriched genes in the lymphohematopoietic tissues. a) Dark blue nodes represent genes that are group enriched in up to five different tissue types including at least one of the lymphohematopoietic tissues. The light blue nodes represent the total number of highly and moderately tissue enriched genes in each lymphohematopoietic tissue. The size of each blue (dark and light) node is related to the square root of the number of genes within. Black circles represent the four lymphohematopoietic tissues, and grey circles denote other tissue types. b) Names of enriched genes (and enhanced genes in brackets) that are exemplified in the text and shown in Fig. 4. The color codes are equal to the nodes in a). The network plot is limited to include nodes with a minimum of two genes.

Altogether 215 genes were found to be tissue enriched or group enriched in at least one of the four tissues of lymphoid or hematological origin. 149 of these were included in a network analysis performed using Cytoscape 3.0 [15] in order to generate an overview of tissue- and group enriched genes in the individual tissues (the plot only includes group enriched nodes with at least two genes and a maximum of five connections to prevent it from being too complex) (Fig. 3). The total number of tissue enriched genes in bone marrow was 80, while this number for spleen, lymph node and appendix was six, five and two respectively (light blue in the figure). All 215 tissue and group enriched as well as enhanced genes are listed in S2 Table.

### Immunohistochemical visualization and characterization of enriched gene expression

Using RNAseq to identify genes with a unique expression in these four lymphohematopoietic tissues is complicated by the fact that immune cells, of both lymphocytic and myelocytic origin, are also naturally occurring in many other peripheral tissues. This circumstance is particularly evident for secondary lymphoid tissues, for which very few genes with a distinct tissue-characteristic expression were identified.

**Figure 4:**
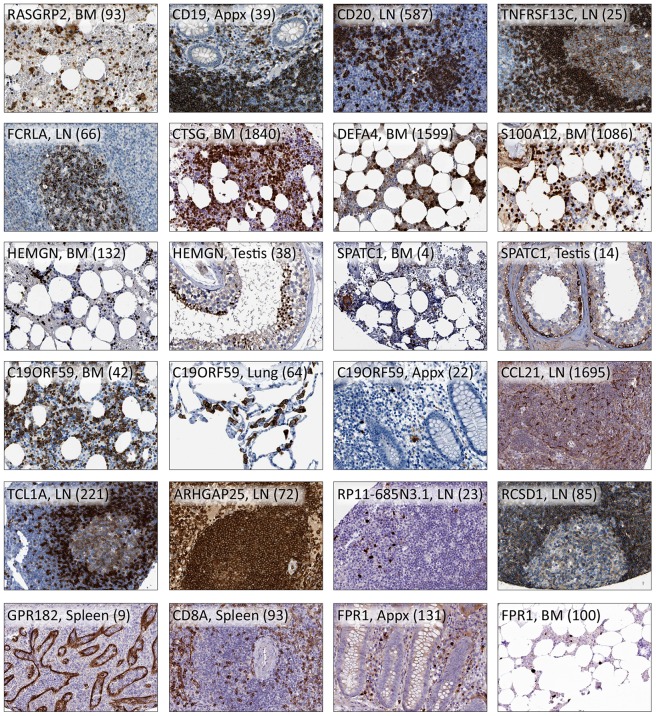
Immunohistochemical staining of protein expression. The staining patterns of 19 proteins transcribed from genes in the enriched or enhanced categories are exemplified here in one to three tissue types. The gene name, tissue type and FPKM-value in brackets are shown for each image. BM: bone marrow; LN: lymph node; Appx: appendix.

The analyses on tissue homogenates still poses a valuable strategy for large scale determination of expression patterns, and in combination with IHC it offers the possibility to identify immune-specific gene expressions. Proteomic data generated using IHC within the Human Protein Atlas initiative [4], [16], [17], was used for visualization of corresponding protein expression in human tissues. Protein profiles using IHC were available for 166 out of 215 genes with tissue/group enriched mRNA expression and 340 out of 478 genes that were enhanced in the lymphohematopoietic tissues. The proteins encoded by many of these genes have known functions in the immune system, e.g. *CD19*, *CD20* (*MS4A1*) or constitute well known cytokines, whereas some are less described (*SPATC1*, *ARHGAP25* and *RCSD1*,) or uncharacterized (*C19ORF59* and *AC233263.1*). The protein expressions of above-mentioned genes are illustrated in Fig. 4.

Due to the large amount of data generated, the following section aims to merely exemplify proteins with an enriched or enhanced expression in lymphohematopoietic tissues identified with the large scale set-up described in this paper. The examples aim to visualize the additional *in situ* information generated by IHC, which is critical when analyzing differentially expressed genes within complex tissues. One prerequisite for inclusion of a protein in the manuscript was therefore the availability of an IHC staining using a stringently validated antibody that supports the RNA-data. The specific examples have been chosen to illustrate i) well-characterized proteins, with known functions in lymphohematopoietic cell types, to validate the set up of the study, as well as ii) previously uncharacterized proteins, to exemplify the potential of the set up to pick up potential proteins of interest for further studies.

#### Enriched in all four lymphohematopoietic tissues

Three genes, *FAM129C*, *TMC8* and *RASGRP2*, were found to be group enriched in the four tissues (Fig. 3). Matching protein profile was available for one of them, *RASGRP2*, which codes for a protein important for platelet function [18]. Furthermore, the RASGRP2 protein has been suggested to play a role in the regulation of neutrophil chemotaxis [19]. Immunohistochemical staining using the antibody HPA015667 shows staining in bone marrow (Fig. 4) and of immune cells in the three lymphatic tissues (mainly mantle zone), as well as in e.g. colon. It also shows strong staining in skeletal muscle, however protein expression in skeletal muscle could not be validated on transcript level due to the lack of RNA data in this tissue.

#### Enriched in the three secondary lymphatic tissues

Sixteen genes were found to be group enriched in appendix, lymph node and spleen, e.g. *CD19*, *CD20* (*MS4A1*), *TNFRSF13C* and *FCRLA*, which are all known to be expressed by B-lymphocytes [20]–[24]. TNFRSF13C, known as B-cell activating factor (BAFF), and FCRLA exemplify protein markers that distinguish two subset of B-cells, as immunohistochemically shown by their distinct cellular distribution in lymph node (Fig. 4). TNFRSF13C is required for the successful maturation and survival of B-cells [25], [26], and is expressed mainly in mantle zone B-cells, while FCRLA-positive cells are primarily found in the germinal center centroblasts [24]. All four markers are included in Fig. 4.

#### Enriched in bone marrow

Bone marrow was the tissue with the highest number of enriched genes. Twenty highly and 60 moderately tissues enriched genes, as well as 57 group enriched genes were found for bone marrow. Most of these are previously well-studied genes, but several uncharacterized genes were also found. Furthermore, 164 genes were found to have an enhanced expression in bone marrow. The relatively great number of genes with an enriched or enhanced expression in bone marrow may reflect the fact that bone marrow represents the sole tissue for generation of all hematopoietic cell linages. It thereby displays the widest repertoire of genes regulating hematopoiesis, especially those genes expressed in the prominent erythro- and myelopoeses.

*CTSG* and *DEFA4* are two of the genes with the highest expression in bone marrow (FPKM 1,840 and 1,599), and they were also categorized as highly enriched. The protein cathepsin G is found in the azurophil granules of neutrophils and has bactericidal properties [27]. Alpha-defensins are a group of proteins also expressed by neutrophils and involved in the defense against bacteria [28], [29]. Defensin alpha 4 has been found to be much more potent against some bacteria compared to the other human defensins [30]. Protein profiles for *CTSG* and *DEFA4* show strong staining of granulocytes (exemplified by bone marrow in Fig. 4).

Also tissue enriched in bone marrow with a high FPKM value (1,086) was *S100A12*, a gene encoding a calcium-binding pro inflammatory protein predominantly secreted by granulocytes. The protein S100A12 has in recent years been suggested to constitute a valuable serum marker for inflammation [31]. Strong and distinct IHC positivity was seen in bone marrow (Fig. 4), spleen and lymph node.

Among the genes enriched in bone marrow we found seven genes also displaying an enriched expression in testis, and two examples of these were *HEMGN* and *SPATC1*. The protein Hemogen is fairly well characterized, and appears to be primarily expressed in immature precursors, and to be involved in hematopoietic differentiation [32]. However, an mRNA isoform different from that found in hematopoietic cells has been identified in round spermatids of testis [33]. Furthermore, the chicken homologue of *HEMGN* has been shown to be a transcription factor specifically involved in sex determination [34]. These findings support our mRNA-data, which shows elevated transcription of *HEMGN* in bone marrow and testis (FPKM 132 and 38 respectively; Fig. 4), compared to other tissues. IHC staining of the protein, Hemogen, is in concordance with the RNA data and literature, showing strong staining of a subset of cells in bone marrow, indicative of erythropoietic cells, and spermatids in testis (Fig. 4). Spermatogenesis and centriole associated 1 (SPATC1) is a protein about which not much is known, although mRNA expression has been found to be pronounced in testis (bone marrow not analyzed), and the SPATC1 has been detected in sperms where it surrounds the intact proximal centriole [35]. IHC using antibody HPA040220 shows a cytoplasmic staining in bone marrow (e.g. megakaryocytes) and in testis, mostly pronounced in spermatogonia, supportive of our RNAseq data (FPKM 4 and 14 respectively; Fig. 4). However, it also shows staining in some other tissues.

An example of a fairly uncharacterized gene that displayed a group enriched expression in bone marrow, along with lung and appendix, is *C19ORF59* (Fig. 4). This gene encodes a single-pass transmembrane protein that was first identified in human mast cells [36]. Our RNAseq data was supported by IHC, showing cytoplasmic staining in alveolar macrophages, and also revealing expression in macrophages of other tissues. Mast cells and macrophages both develop from the myeloid progenitor and it is therefore possible that the two cell types express this gene. A large population of cells, indicative of granulopoietic cells, in bone marrow was stained, which is consistent with our RNA-data. Granulocytes were also stained in secondary lymphoid tissues and in the GI-tract. Immunohistochemical analysis of a large panel of human cell lines revealed a distinct expression in myeloid cell lines HL-60 (acute promyelocytic leukemia cell line) [37], [38], THP-1 (acute monocytic leukemia cell line) [39] and U-937 (myelomonocytic sarcoma cell line) [40], suggesting a function important for cells of monocytic lineage. A weak IHC positivity was however also seen in HMC-1 (mast cell leukemia cell line) supporting earlier results that suggests the protein product of *C19ORF59* to be involved in regulating mast cell differentiation or immune responses [36].

#### Enriched and enhanced in lymph node

Due to the inherent presence of infiltrating lymphocytes in most normal human tissues, only few genes are categorized as tissue enriched in lymph node.

Five genes were found to be moderately tissue enriched and 44 genes were group enriched, and 183 displayed an enhanced expression. RNA-data for all enriched genes can be found in S2 Table, and matching protein characterization for *CCL21* is shown in Fig. 4.

The protein CCL21 is expressed in secondary lymphoid organs (e.g. lymph node) and is known to attract T- and B-cells and dendritic cells via their chemokine receptor (CCR7) [41], [42]. Our RNAseq-data showed a moderately enriched expression in lymph node, which is in concordance with IHC showing staining of dendritic cells outside the germinal centers (HPA051210, pending publishing; Fig. 4). *CCL21* is also the gene with the highest FPKM-value (1,695) among the tissues/group enriched and enhanced genes for lymph node.

*TCL1A* displayed an enhanced expression in lymph node, and IHC staining using antibody HPA016604 supports the RNA-data, showing positivity in lymphocytes mainly in B-cell areas of the lymph node (Fig. 4) and other secondary lymphoid organs. Dysregulation of *TCL1A* leading to overexpression of the protein is associated with the development of mature T cell leukemia, which has been shown using transgenic mice [43], and chronic lymphocytic leukemia that has been studied both in patient cohorts [44] and in mouse models [45].

*ARHGAP25* was identified and characterized in 2004 [46]. The protein belongs to a family of Rho GTPase-modulating proteins, and it has been shown to be specifically expressed in hematopoietic cells, and to act as a negative regulator of phagocytosis [47]. *ARHGAP25* displayed an enhanced RNA-expression in lymph node, as well as being expressed at relatively high levels in the other three lymphohematopoietic tissues. The expression pattern was further supported by IHC analysis, showing strong staining in all four lymphoid tissues and in bone marrow, as exemplified with lymph node in Fig. 4.

Also enhanced in lymph node was gene *RP11-685N3.1* (earlier AC233263.1), about which nothing is known. Transcript data displays enhanced expression also in colon, appendix and urinary bladder, and immunohistochemical data (antibody HPA047702) supports this with distinct staining in a subset of immune cells in these tissues. Fig. 4 shows the staining of the protein RP11-685N3.1 in lymph node.

*RCSD1*, enhanced in lymph node, has recently been identified as a novel gene fusion partner of the *ABL1* gene in B-lymphoblastic leukemia [48]. According to our transcriptional and immunohistochemical data *RCSD1* is expressed in all four lymphohematopoietic tissues. Fig. 4 shows strong cytoplasmic staining of the corresponding protein in the marginal zone and weaker staining in the germinal center of lymph node. Cell lines of lymphoid and myeloid origin in our panel were also positive.

#### Enriched and enhanced in spleen

Six genes were defined as moderately tissue enriched in spleen, 57 were group enriched, and 129 genes showed an enhanced gene expression in spleen. Several of the spleen characteristic genes are previously uncharacterized.

The transcription of *GPR182* was moderately enriched in spleen, suggesting a function in the immune system. However, IHC showed expression of the protein in sinus endothelium, with the strongest staining seen in spleen (Fig. 4) and lymph node. This exemplifies the necessity of complementing RNAseq data on tissue samples with the use of *in situ* methods revealing information on a cellular level, especially for previously uncharacterized genes. The G protein-coupled receptor GPR182 is fairly uncharacterized, however it has been shown to be highly expressed in endothelial cells of mouse embryos [49] and in *in vitro*-cultured tumor-specific endothelial cells [50], supporting our antibody staining of sinus endothelium seen in spleen and lymph node.

*CD8A* codes for the alpha chain of the cell surface glycoprotein CD8, a co-receptor to the T-cell receptor found on most cytotoxic T-lymphocytes. RNA-data implies an enhanced expression in spleen, which is in concordance with IHC staining (Fig. 4) of the protein (HPA037756 and CAB000012). Positive lymphocytes are also seen in the other three lymphohematopoietic organs and in some other tissues. Furthermore, vessels in spleen were strongly stained, which might contribute to the elevated expression here. CD8-positivity in splenic sinus endothelium has been described before [51].

#### Enriched and enhanced in appendix

In appendix, two genes were moderately tissue enriched (not confirmed by IHC), 56 were group enriched, and 73 genes displayed an enhanced expression. A few uncharacterized genes were found in these categories.

One of the most highly expressed genes found in appendix was *FPR1* (FPKM 131), which is categorized as enhanced in appendix as well as in bone marrow (FPKM 100). *FPR1* codes for a G protein-coupled receptor protein expressed by e.g. neutrophils, and plays a role in chemotaxis, phagocytosis and generation of reactive oxygen species [52]. The IHC staining supports our RNA-data, showing strong staining in a cell population indicative of phagocytes in bone marrow and appendix (Fig. 4), as well as in several other tissues. Positivity was also seen in high endothelial venules of lymph node, which are vessels in the paracortex where cross over of T-cells takes place.

## Conclusions

Transcriptomics of high quality human tissue samples, evaluated by pathologists for representativity, were analyzed to identify expression of tissue-specific genes in 27 different tissues [11], including four lymphohematopoietic tissue types. Most genes were expressed in many or all tissues, although 3.5% (693) showed an enriched or enhanced expression in one or more of the hematological tissues. Several of these encode previously uncharacterized proteins, and therefore constitute possible new targets for research. Here, we present examples of both well-known and uncharacterized genes with elevated expression in the lymphohematopoietic tissues. However, all RNAseq data for the 20,050 genes analyzed are publically available at the ArrayExpress Database [53] under the accession number E-MTAB-1733. Furthermore, the Human Protein Atlas was used as a resource to compare RNAseq data with IHC on tissues, providing information about the expression pattern on a cellular level. Version 12 of the freely available protein atlas [4] offers protein profiling of 16,621 genes on 185 tissue and cell line samples. In combination, these two high throughput strategies on RNA and protein offer a method to find potentially interesting gene expressions for certain tissue-/cell types.

## Methods

### Tissue and cell samples

Fresh frozen material from 27 different morphologically normal tissues was included in this study (S1 Table). Tissues were obtained from the Uppsala Biobank with ethical approval of the Regional Ethical Review Board in Uppsala (2011/473). With the number of biological replicates for each organ ranging from 1–5, overall samples from 95 different individuals were analyzed. In this study in particular the expression profile of 3–5 replicate samples each from bone marrow, spleen, lymph node and appendix were profiled and compared to other organs (Table 3). All samples included in the study were examined by a pathologist in order to verify representativity. Spleen and lymph node samples were all assessed as morphologically normal. Appendix tissue represented cases of appendicitis, and acute inflammatory reactions were evident. Bone marrow samples were evaluated by pathologist to contain morphologically normal cells, with a hematopoietic component of approximately 52% myelopoietic cells, 32% erythropoietic cells, 11% lymphocytes, and 5% monocytes. The bone marrow samples used in this study were Ficoll separated preparations, in which the non-hematopoietic components of stroma, adipose cells and vessels as well as large portions of the erythrocytes and granulocytes have been removed. Lymph node, spleen and appendix samples were embedded into Optimal Cutting Temperature (OCT) medium and stored at −80°C until sectioned and analyzed. Bone marrow samples were stored as pellets in −80°C until analyzed.

**Table 3:** Tissue samples included for RNA expression profiling.

	Lymph node	Appendix	Spleen	Bone marrow
Lymphopoietic cells	85–95	65–75	40	11
Macrophages/monocytes	<1	<1	30–35	5
Myelopoietic cells	<1	<1	<1	52
Erythropoietic cells*	-	-	-	32
Endothelial cells	<1	<1	15	-
Other cell types**	*5–15*	*25–35*	*10–15*	-

The estimated percentages of cells are given for each tissue type. In bone marrow, cells of erythropoietic origin are mostly immature and nucleated precursors. * In secondary lymphoid organs, fully maturated erythrocytes are not included, since they are unlikely to contribute to the transcriptomic profile. ** Other cell types refer to adipocytes, fibroblasts, smooth muscle cells and glandular cells.

### Transcriptomic Profiling by RNA-sequencing

Next generation mRNA-sequencing was performed as earlier described [54]. In short, frozen 4 µm sections were cut from each tissue block, and subsequently stained with hematoxylin and eosin and examined by a pathologist to ensure representative morphology. For RNA extraction, six 10 µm sections were homogenized and total RNA extracted using the RNeasy Mini Kit (Qiagen, Hilden, Germany) according to protocol provided by the manufacturer. The quality of the extracted RNA was determined on either an Experion automated electrophoresis system (Bio-Rad Laboratories, Hercules, CA, USA) using a standard-sensitivity RNA chip or an Agilent 2100 Bioanalyzer system (Agilent Biotechnologies, Palo Alto, USA) using an RNA 6000 Nano Labchip Kit. Samples with RNA Integrity Number >7.5 were included the study. Library preparation was performed using the TruSeq RNA Sample Prep Kit (Illumina, San Diego, CA, USA) following the manufacturer's instructions. Next generation mRNA-sequencing was performed on Illumina HiSeq2000 and 2500 machines (Illumina) using a standard Illumina RNAseq protocol with a read length of 2×100 bases.

### Data Analysis

Raw reads obtained from the sequencing system were trimmed for low quality ends using a software called Sickle [55]. A phred quality threshold of 20 was applied. Only reads >54 bp after trimming were included. Processed reads were mapped to the version GRCh37 of the human genome using Tophat version 2.0.3 [56]. Potential PCR duplicates were eliminated through application of the MarkDuplicates module of Picard version 1.77 [57]. To obtain quantification scores for all human genes, fragments per kilobase of exon model per million mapped reads (FPKM) values were calculated using Cufflinks v2.0.2 [58]. This software corrects for transcript length and the total number of mapped reads from the library to compensate for differences in read depths in different samples. Gene models from Ensembl build 69 [59] were used in Cufflinks. All data were analyzed using the R Statistical Environment [60] with the addition of the ‘gplots’ package [61]. A network analysis was performed using Cytoscape version 3.0 [15]. For analyses requiring a log2-scale of the data, pseudo-counts of +1 were added to the data set. A GO [62] analysis was performed using the GOrilla tool [63] in order to determine overrepresented GO categories in certain sets of genes. A list of all genes analyzed in this study was used as the background list in GOrilla.

### Barcode “Leakage”

In the present global study, we observed approximately 0.1% of misidentified reads for samples that were sequenced in a multiplexed way. This has been previously observed [64], and is known to introduce a minor bias to the data. However, since most analyses were based on relative differences (fold-changes) compared the tissue with highest expression, the observed leakage of 0.1% was not considered to substantially alter the results.

### Categorization of gene expression patterns

For each tissue, the average FPKM value for all replicates was used as a measure of gene expression level. A cut-off value of FPKM = 1 was set as the limit of detection. Each of the 20,050 genes was classified into one of seven categories based on the expression levels over all 27 tissues (Table 1).

### Antibody-based Tissue Profiling

Large-scale protein profiling within the Human Protein Atlas project has been previously described [1], [65]. In brief, antibody-based protein expression profiling was performed on archival material of formalin fixed paraffin embedded (FFPE) material assembled in tissue microarrays (TMAs). Ethical approval was granted by the Regional Ethical Review Board in Uppsala (Reference Ups 02-577). TMAs were generated containing triplicate 1 mm cores from each of 46 different types of normal tissue and duplicate 1 mm cores from each of 216 different cancer tissues representing the 20 most common forms of human cancer. Altogether one section from each of eight TMA-designs was stained using IHC. All IHC-stained tissues were then transformed into digitalized images using The Aperio ScanScope XT Slide Scanner (Aperio Technologies, Vista, CA) using a 20x objective. The outcome of IHC stainings was evaluated manually and scored by certified pathologists using a web-based annotation system. The staining intensity was annotated as negative, low, medium or high. The fraction of stained cells was graded as >75%, 25%–75%, <25% or “rare”. Detailed staining results for all antibodies included in the study can be accessed in the publicly available Human Protein atlas [4]. Antibody ID-numbers can be found in S2 Table.

### Availability of supporting data

The data set supporting the results of this article is available in the version 13 of the Human Protein Atlas, and the primary data (reads) are available through the Array Express Archive [53] under the accession number E-MTAB-1733.

## Supporting Information

S1 Fig **Immunohistochemical staining of CEACAM5 and GAL.** The staining patterns of CEACAM5 in colon and colorectal cancer, and of GAL in colon are shown. CRC: colorectal cancer.
(TIF)

S2 Fig **Pie charts comparing the gene expression between the lymphohematopoietic tissues.** The percentages of genes expressed within the different categories of expression are shown for appendix, bone marrow, lymph node and spleen.
(TIF)

S1 Table Fresh frozen material from 27 different morphologically normal* tissues was included in this study. * All cases of appendix represent cases of appendicitis.
(XLSX)

S2 Table **All genes with elevated expression in the four lymphohematopoietic tissues (93 tissue enriched, 122 group enriched, and 478 enhanced genes).** Genes in bold are exemplified in the paper and shown in Fig. 4.
(XLSX)

## References

[1] Ponten F, Jirstrom K, Uhlen M (2008) The Human Protein Atlas–a tool for pathology. J Pathol 216:387–393. 18853439 10.1002/path.2440

[2] Uhlen M, Bjorling E, Agaton C, Szigyarto CA, Amini B, et al (2005) A human protein atlas for normal and cancer tissues based on antibody proteomics. Mol Cell Proteomics 4:1920–1932. 16127175 10.1074/mcp.M500279-MCP200

[3] Bain BJ, Clark DM, Lampert IA (1996) Bone marrow pathology. Oxford; Cambridge, Mass., USA: Blackwell Science. viii, 328 p. p.

[4] (2014) The Human Protein Atlas.

[5] UniProt C (2013) Update on activities at the Universal Protein Resource (UniProt) in 2013. Nucleic Acids Res 41:D43–47. 23161681 10.1093/nar/gks1068 PMC3531094

[6] Petryszak R, Burdett T, Fiorelli B, Fonseca NA, Gonzalez-Porta M, et al (2014) Expression Atlas update–a database of gene and transcript expression from microarray- and sequencing-based functional genomics experiments. Nucleic Acids Res 42:D926–932. 24304889 10.1093/nar/gkt1270 PMC3964963

[7] Su AI, Wiltshire T, Batalov S, Lapp H, Ching KA, et al (2004) A gene atlas of the mouse and human protein-encoding transcriptomes. Proc Natl Acad Sci U S A 101:6062–6067. 15075390 10.1073/pnas.0400782101 PMC395923

[8] Wu C, Orozco C, Boyer J, Leglise M, Goodale J, et al (2009) BioGPS: an extensible and customizable portal for querying and organizing gene annotation resources. Genome Biol 10:R130. 19919682 10.1186/gb-2009-10-11-r130 PMC3091323

[9] Parkinson H, Sarkans U, Shojatalab M, Abeygunawardena N, Contrino S, et al (2005) ArrayExpress–a public repository for microarray gene expression data at the EBI. Nucleic Acids Res 33:D553–555. 15608260 10.1093/nar/gki056 PMC540010

[10] Krupp M, Marquardt JU, Sahin U, Galle PR, Castle J, et al (2012) RNA-Seq Atlas–a reference database for gene expression profiling in normal tissue by next-generation sequencing. Bioinformatics 28:1184–1185. 22345621 10.1093/bioinformatics/bts084

[11] Fagerberg L, Hallstrom BM, Oksvold P, Kampf C, Djureinovic D, et al (2014) Analysis of the Human Tissue-specific Expression by Genome-wide Integration of Transcriptomics and Antibody-based Proteomics. Mol Cell Proteomics 13:397–406. 24309898 10.1074/mcp.M113.035600 PMC3916642

[12] Lee YN (1978) Carcinoembryonic antigen in patients with breast or colon cancer. West J Med 129:374–380. 364839 PMC1238388

[13] Mechenthaler I (2008) Galanin and the neuroendocrine axes. Cell Mol Life Sci 65:1826–1835. 18500643 10.1007/s00018-008-8157-4 PMC11131683

[14] Hammond ME, Lapointe GR, Feucht PH, Hilt S, Gallegos CA, et al (1995) IL-8 induces neutrophil chemotaxis predominantly via type I IL-8 receptors. J Immunol 155:1428–1433. 7636208

[15] Shannon P, Markiel A, Ozier O, Baliga NS, Wang JT, et al (2003) Cytoscape: a software environment for integrated models of biomolecular interaction networks. Genome Res 13:2498–2504. 14597658 10.1101/gr.1239303 PMC403769

[16] Ponten F, Schwenk JM, Asplund A, Edqvist PH (2011) The Human Protein Atlas as a proteomic resource for biomarker discovery. J Intern Med 270:428–446. 21752111 10.1111/j.1365-2796.2011.02427.x

[17] Uhlen M, Oksvold P, Fagerberg L, Lundberg E, Jonasson K, et al (2010) Towards a knowledge-based Human Protein Atlas. Nat Biotechnol 28:1248–1250. 21139605 10.1038/nbt1210-1248

[18] Crittenden JR, Bergmeier W, Zhang Y, Piffath CL, Liang Y, et al (2004) CalDAG-GEFI integrates signaling for platelet aggregation and thrombus formation. Nat Med 10:982–986. 15334074 10.1038/nm1098

[19] Carbo C, Duerschmied D, Goerge T, Hattori H, Sakai J, et al (2010) Integrin-independent role of CalDAG-GEFI in neutrophil chemotaxis. J Leukoc Biol 88:313–319. 20413728 10.1189/jlb.0110049 PMC2908939

[20] Tedder TF, Isaacs CM (1989) Isolation of cDNAs encoding the CD19 antigen of human and mouse B lymphocytes. A new member of the immunoglobulin superfamily. J Immunol 143:712–717. 2472450

[21] Tedder TF, Streuli M, Schlossman SF, Saito H (1988) Isolation and structure of a cDNA encoding the B1 (CD20) cell-surface antigen of human B lymphocytes. Proc Natl Acad Sci U S A 85:208–212. 2448768 10.1073/pnas.85.1.208 PMC279513

[22] Thompson JS, Bixler SA, Qian F, Vora K, Scott ML, et al (2001) BAFF-R, a newly identified TNF receptor that specifically interacts with BAFF. Science 293:2108–2111. 11509692 10.1126/science.1061965

[23] Mechetina LV, Najakshin AM, Volkova OY, Guselnikov SV, Faizulin RZ, et al (2002) FCRL, a novel member of the leukocyte Fc receptor family possesses unique structural features. Eur J Immunol 32:87–96. 11754007 10.1002/1521-4141(200201)32:1<87::AID-IMMU87>3.0.CO;2-#

[24] Facchetti F, Cella M, Festa S, Fremont DH, Colonna M (2002) An unusual Fc receptor-related protein expressed in human centroblasts. Proc Natl Acad Sci U S A 99:3776–3781. 11891275 10.1073/pnas.022042699 PMC122600

[25] Schiemann B, Gommerman JL, Vora K, Cachero TG, Shulga-Morskaya S, et al (2001) An essential role for BAFF in the normal development of B cells through a BCMA-independent pathway. Science 293:2111–2114. 11509691 10.1126/science.1061964

[26] Schneider P, MacKay F, Steiner V, Hofmann K, Bodmer JL, et al (1999) BAFF, a novel ligand of the tumor necrosis factor family, stimulates B cell growth. J Exp Med 189:1747–1756. 10359578 10.1084/jem.189.11.1747 PMC2193079

[27] Thorne KJ, Oliver RC, Barrett AJ (1976) Lysis and killing of bacteria by lysosomal proteinases. Infect Immun 14:555–563. 971964 10.1128/iai.14.2.555-563.1976 PMC420918

[28] Ganz T, Selsted ME, Szklarek D, Harwig SS, Daher K, et al (1985) Defensins. Natural peptide antibiotics of human neutrophils. J Clin Invest 76:1427–1435. 2997278 10.1172/JCI112120 PMC424093

[29] Ericksen B, Wu Z, Lu W, Lehrer RI (2005) Antibacterial activity and specificity of the six human {alpha}-defensins. Antimicrob Agents Chemother 49:269–275. 15616305 10.1128/AAC.49.1.269-275.2005 PMC538877

[30] Wilde CG, Griffith JE, Marra MN, Snable JL, Scott RW (1989) Purification and characterization of human neutrophil peptide 4, a novel member of the defensin family. J Biol Chem 264:11200–11203. 2500436

[31] Meijer B, Gearry RB, Day AS (2012) The role of S100A12 as a systemic marker of inflammation. Int J Inflam 2012:907078. 22811950 10.1155/2012/907078 PMC3395136

[32] Yang LV, Nicholson RH, Kaplan J, Galy A, Li L (2001) Hemogen is a novel nuclear factor specifically expressed in mouse hematopoietic development and its human homologue EDAG maps to chromosome 9q22, a region containing breakpoints of hematological neoplasms. Mech Dev 104:105–111. 11404085 10.1016/s0925-4773(01)00376-8

[33] Yang LV, Heng HH, Wan J, Southwood CM, Gow A, et al (2003) Alternative promoters and polyadenylation regulate tissue-specific expression of Hemogen isoforms during hematopoiesis and spermatogenesis. Dev Dyn 228:606–616. 14648837 10.1002/dvdy.10399

[34] Nakata T, Ishiguro M, Aduma N, Izumi H, Kuroiwa A (2013) Chicken hemogen homolog is involved in the chicken-specific sex-determining mechanism. Proc Natl Acad Sci U S A 110:3417–3422. 23401550 10.1073/pnas.1218714110 PMC3587191

[35] Goto M, O′Brien DA, Eddy EM (2010) Speriolin is a novel human and mouse sperm centrosome protein. Hum Reprod 25:1884–1894. 20542897 10.1093/humrep/deq138 PMC2907228

[36] Li K, Wang SW, Li Y, Martin RE, Li L, et al (2005) Identification and expression of a new type II transmembrane protein in human mast cells. Genomics 86:68–75. 15953541 10.1016/j.ygeno.2005.03.006

[37] Gallagher R, Collins S, Trujillo J, McCredie K, Ahearn M, et al (1979) Characterization of the continuous, differentiating myeloid cell line (HL-60) from a patient with acute promyelocytic leukemia. Blood 54:713–733. 288488

[38] Collins SJ, Gallo RC, Gallagher RE (1977) Continuous growth and differentiation of human myeloid leukaemic cells in suspension culture. Nature 270:347–349. 271272 10.1038/270347a0

[39] Tsuchiya S, Yamabe M, Yamaguchi Y, Kobayashi Y, Konno T, et al (1980) Establishment and characterization of a human acute monocytic leukemia cell line (THP-1). Int J Cancer 26:171–176. 6970727 10.1002/ijc.2910260208

[40] Sundstrom C, Nilsson K (1976) Establishment and characterization of a human histiocytic lymphoma cell line (U-937). Int J Cancer 17:565–577. 178611 10.1002/ijc.2910170504

[41] Gunn MD, Tangemann K, Tam C, Cyster JG, Rosen SD, et al (1998) A chemokine expressed in lymphoid high endothelial venules promotes the adhesion and chemotaxis of naive T lymphocytes. Proc Natl Acad Sci U S A 95:258–263. 9419363 10.1073/pnas.95.1.258 PMC18193

[42] Saeki H, Moore AM, Brown MJ, Hwang ST (1999) Cutting edge: secondary lymphoid-tissue chemokine (SLC) and CC chemokine receptor 7 (CCR7) participate in the emigration pathway of mature dendritic cells from the skin to regional lymph nodes. J Immunol 162:2472–2475. 10072485

[43] Virgilio L, Lazzeri C, Bichi R, Nibu K, Narducci MG, et al (1998) Deregulated expression of TCL1 causes T cell leukemia in mice. Proc Natl Acad Sci U S A 95:3885–3889. 9520462 10.1073/pnas.95.7.3885 PMC19932

[44] Aggarwal M, Villuendas R, Gomez G, Rodriguez-Pinilla SM, Sanchez-Beato M, et al (2009) TCL1A expression delineates biological and clinical variability in B-cell lymphoma. Mod Pathol 22:206–215. 18820675 10.1038/modpathol.2008.148

[45] Bichi R, Shinton SA, Martin ES, Koval A, Calin GA, et al (2002) Human chronic lymphocytic leukemia modeled in mouse by targeted TCL1 expression. Proc Natl Acad Sci U S A 99:6955–6960. 12011454 10.1073/pnas.102181599 PMC124510

[46] Katoh M, Katoh M (2004) Identification and characterization of ARHGAP24 and ARHGAP25 genes in silico. Int J Mol Med 14:333–338. 15254788

[47] Csepanyi-Komi R, Sirokmany G, Geiszt M, Ligeti E (2012) ARHGAP25, a novel Rac GTPase-activating protein, regulates phagocytosis in human neutrophilic granulocytes. Blood 119:573–582. 22096251 10.1182/blood-2010-12-324053

[48] De Braekeleer E, Douet-Guilbert N, Le Bris MJ, Berthou C, Morel F, et al (2007) A new partner gene fused to ABL1 in a t(1;9)(q24;q34)-associated B-cell acute lymphoblastic leukemia. Leukemia 21:2220–2221. 17541395 10.1038/sj.leu.2404773

[49] Takase H, Matsumoto K, Yamadera R, Kubota Y, Otsu A, et al (2012) Genome-wide identification of endothelial cell-enriched genes in the mouse embryo. Blood 120:914–923. 22535667 10.1182/blood-2011-12-398156

[50] Xiao L, Harrell JC, Perou CM, Dudley AC (2013) Identification of a stable molecular signature in mammary tumor endothelial cells that persists in vitro. Angiogenesis 10.1007/s10456-013-9409-y PMC4029871 24257808

[51] Steininger H, Pfofe D, Marquardt L, Sauer H, Markwat R (2004) Isolated diffuse hemangiomatosis of the spleen: case report and review of literature. Pathol Res Pract 200:479–485. 15310152 10.1016/j.prp.2004.04.004

[52] Ye RD, Boulay F, Wang JM, Dahlgren C, Gerard C, et al (2009) International Union of Basic and Clinical Pharmacology. LXXIII. Nomenclature for the formyl peptide receptor (FPR) family. Pharmacol Rev 61:119–161. 19498085 10.1124/pr.109.001578 PMC2745437

[53] (2014) The ArrayExpress Database.

[54] Fagerberg L, Hallstrom BM, Oksvold P, Kampf C, Djureinovic D, et al (2013) Analysis of the human tissue-specific expression by genome-wide integration of transcriptomics and antibody-based proteomics. Mol Cell Proteomics 10.1074/mcp.M113.035600 PMC3916642 24309898

[55] (2012) Sickle - A windowed adaptive trimming tool for FASTQ files using quality. https://github.com/najoshi/sickle.

[56] Trapnell C, Pachter L, Salzberg SL (2009) TopHat: discovering splice junctions with RNA-Seq. Bioinformatics 25:1105–1111. 19289445 10.1093/bioinformatics/btp120 PMC2672628

[57] (2012) Picard. http://picardsourceforgenet/.

[58] Trapnell C, Williams BA, Pertea G, Mortazavi A, Kwan G, et al (2010) Transcript assembly and quantification by RNA-Seq reveals unannotated transcripts and isoform switching during cell differentiation. Nat Biotechnol 28:511–515. 20436464 10.1038/nbt.1621 PMC3146043

[59] Flicek P, Amode MR, Barrell D, Beal K, Brent S, et al (2012) Ensembl 2012. Nucleic acids research 40:D84–90. 22086963 10.1093/nar/gkr991 PMC3245178

[60] RCoreTeam (2013) R: A language and environment for statistical computing. R Foundation for Statistical Computing Vienna, Austria. http://www.R-project.org/.

[61] Gregory RW (2012) gplots: Various R programming tools for plotting data. R package version 2.11.0. http://CRANR-projectorg/package=gplots.

[62] Ashburner M, Ball CA, Blake JA, Botstein D, Butler H, et al (2000) Gene ontology: tool for the unification of biology. The Gene Ontology Consortium. Nat Genet 25:25–29. 10802651 10.1038/75556 PMC3037419

[63] Eden E, Navon R, Steinfeld I, Lipson D, Yakhini Z (2009) GOrilla: a tool for discovery and visualization of enriched GO terms in ranked gene lists. BMC Bioinformatics 10:48. 19192299 10.1186/1471-2105-10-48 PMC2644678

[64] Kircher M, Sawyer S, Meyer M (2012) Double indexing overcomes inaccuracies in multiplex sequencing on the Illumina platform. Nucleic Acids Res 40:e3. 22021376 10.1093/nar/gkr771 PMC3245947

[65] Kampf C, Andersson A-C, Wester K, Björling E, Uhlen M, et al (2004) Antibody-based tissue profiling as a tool for clinical proteomics. Clinical Proteomics 1:285–299.

