# Supplementary figures and images for: The Transcriptomic and Proteomic Landscapes of Bone Marrow and Secondary Lymphoid Tissues
Source: PLoS One. 2014 Dec 26;9(12):e115911. doi: 10.1371/journal.pone.0115911 (PMC4277406; doi:10.1371/journal.pone.0115911)

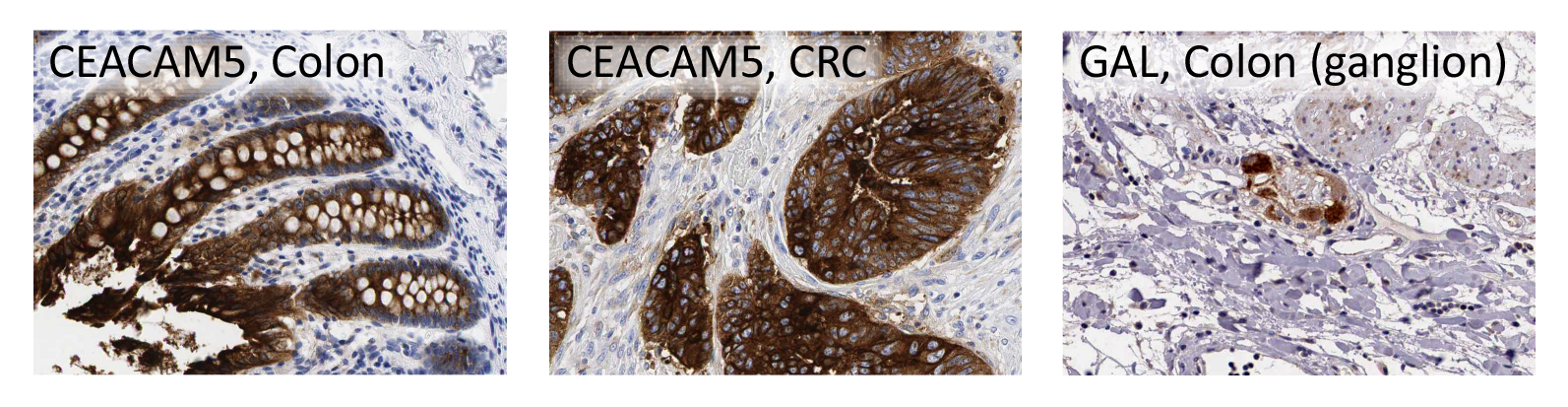

Supplement: S1 Fig — Immunohistochemical staining of CEACAM5 and GAL. The staining patterns of CEACAM5 in colon and colorectal cancer, and of GAL in colon are shown. CRC: colorectal cancer. (TIF) [file pone.0115911.s001.tif]

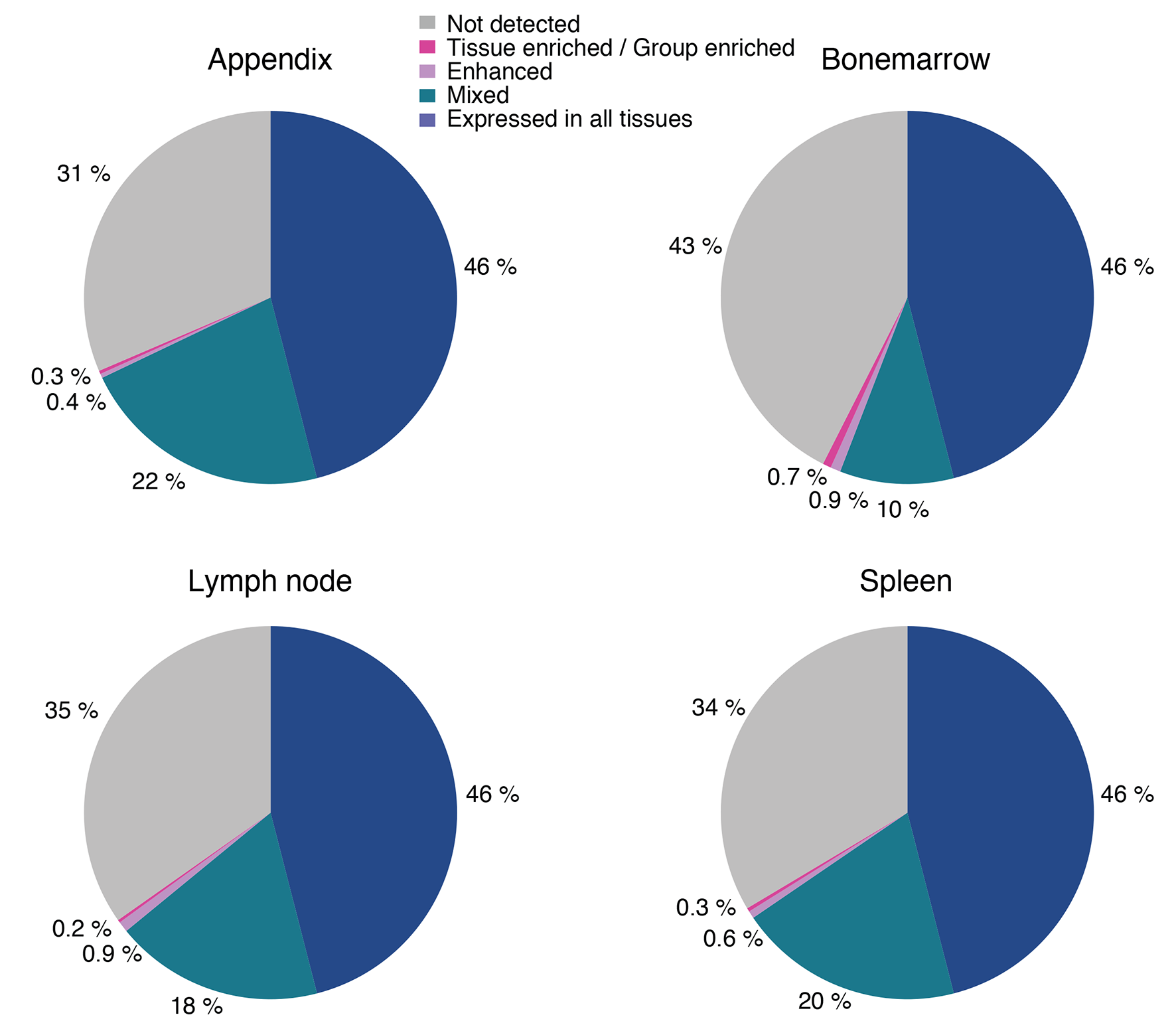

Supplement: S2 Fig — Pie charts comparing the gene expression between the lymphohematopoietic tissues. The percentages of genes expressed within the different categories of expression are shown for appendix, bone marrow, lymph node and spleen. (TIF) [file pone.0115911.s002.tif]
